# Sample changers for direct geometry neutron chopper spectrometers

**DOI:** 10.1038/s41598-025-17049-3

**Published:** 2025-08-29

**Authors:** Matthew B. Stone, Garrett E. Granroth, Daniel M. Pajerowski, Douglas L. Abernathy, David L. Conner, Lisa DeBeer-Schmitt, Victor R. Fanelli, Richard Goyette, Alexander I. Kolesnikov, Rebecca Mills, Mary Odom, Andrey Podlesnyak, Christopher Schmitt, Todd E. Sherline, Landon Solomon, John F. Wenzel

**Affiliations:** 1 Neutron Scattering Division, Oak Ridge National Laboratory, Oak Ridge, 37831 TN USA; 2 Neutron Technology Division, Oak Ridge National Laboratory, Oak Ridge, 37831 TN USA; 3 Kuori Oy, Espoo, Finland

**Keywords:** Condensed-matter physics, Techniques and instrumentation

## Abstract

The advent of higher flux neutron sources has made the use of sample changers appropriate across the instrument suites at neutron scattering facilities. We examine the efficiency, design, and operation of two sample changers used at the thermal chopper spectrometers at the Spallation Neutron Source. We also present case studies of sample holders designed to accommodate multiple single crystal or powder samples for the Cold Neutron Chopper Spectrometer at the Spallation Neutron Source. [This manuscript has been authored by UT-Battelle, LLC under Contract No. DE-AC05-00OR22725 with the U.S. Department of Energy. The United States Government retains and the publisher, by accepting the article for publication, acknowledges that the United States Government retains a non-exclusive, paid-up, irrevocable, world-wide license to publish or reproduce the published form of this manuscript, or allow others to do so, for United States Government purposes. The Department of Energy will provide public access to these results of federally sponsored research in accordance with the DOE Public Access Plan (http://energy.gov/downloads/doe-public-access-plan).]

## Introduction

Neutron scattering has long been considered a flux limited technique. This means that the time required to measure sufficient statistics, the so-called counting time, was always much greater than any other time constraint in the experiment. As high flux neutron sources^1–3^ and advanced optics^4–7^ to transport neutrons have become available, times to perform other aspects of experiments, e.g. sample temperature changes or sample changes, begin to significantly impact the number of experiments that can be performed.

In general, an increase of neutron flux for fixed instrumentation can be used in several ways. First, more difficult measurements requiring finer instrumental resolution^8,9^, polarization or other neutron flux limited techniques become more feasible. Second, because the signal from a neutron scattering experiment is directly proportional to the quantity of material in the sample, as flux increases, proportionally smaller sample sizes are able to be measured^10^. This allows rapid neutron scattering measurements of new materials to be performed earlier in their characterization cycle when only small amounts of these materials may be available. Likewise, data collection on traditional sample sizes can be performed faster. Higher neutron flux also allows for viable measurements of sample environments with thicker boundaries, e.g. pressure cells or more complicated magnetic field or cryostat sample environments.

Faster experiments allow sample parameters such as temperature or applied magnetic field to be continuously changed during data collection^11^. Faster experiments also allow for an increase in experimental capacity at a given instrument. In other words, a greater number of samples can be measured at a given neutron scattering instrument in a fixed amount of operational time. The fixed amount of time required to change samples at an instrument, including cooling and heating times now becomes significant and, unfortunately, limits the increase in experimental capacity.

Sample changers are a standard method used at scattering facilities in order to preserve and improve experiment capacity at a given instrument. Sample changers are in regular use at neutron powder diffractometers^12–14^, including high pressure powder neutron diffraction^15^, and small angle neutron scattering (SANS) instruments^16^. Recently, powder sample changers have also been initiated for neutron spectrometers such as at the VISION instrument at the Spallation Neutron Source (SNS)^17^. With the proton power upgrade project at the SNS^18^, investments in sample changers for direct geometry neutron chopper spectrometers have also been made a priority. Further gains in efficiency can also be made through the use of sample cans that are able to contain multiple samples without cross-contamination in scattered neutrons. This manuscript examines neutron scattering sample changers for direct geometry chopper spectrometers as well as the design and use of multi-sample cans to improve the experimental efficiency of these instruments.

Neutron scattering is an especially useful probe of structure and dynamics of materials^19^. One advantage of neutron scattering is that, because the neutron is a weakly interacting probe of matter, samples can be measured within complicated sample environments. Pressure, temperature, and magnetic field sample environments, as well as combinations thereof, are all routinely used at neutron scattering facilities^20–22^. Temperature is likely the most commonly changed sample environment parameter for neutron scattering measurements. The energy scale of epi-thermal, thermal, and cold neutrons as well as the energy scale of the excitations commonly probed with neutron scattering, i.e. phonons and magnons, in condensed matter systems overlaps very well with temperature ranges between $T=0.01$ K and $T=2700$ K. Closed cycle refrigerators (CCR) available from manufacturers are able to routinely operate between $T=5$ K and $T=325$ K without modifications of the cold-head^23^. This range of temperatures is ideal for measuring excitations at thermal and epi-thermal neutron scattering instrumentation which operates with neutron energies between $E\approx 4$ and $E\approx 6000$ meV.

**Fig. 1:**
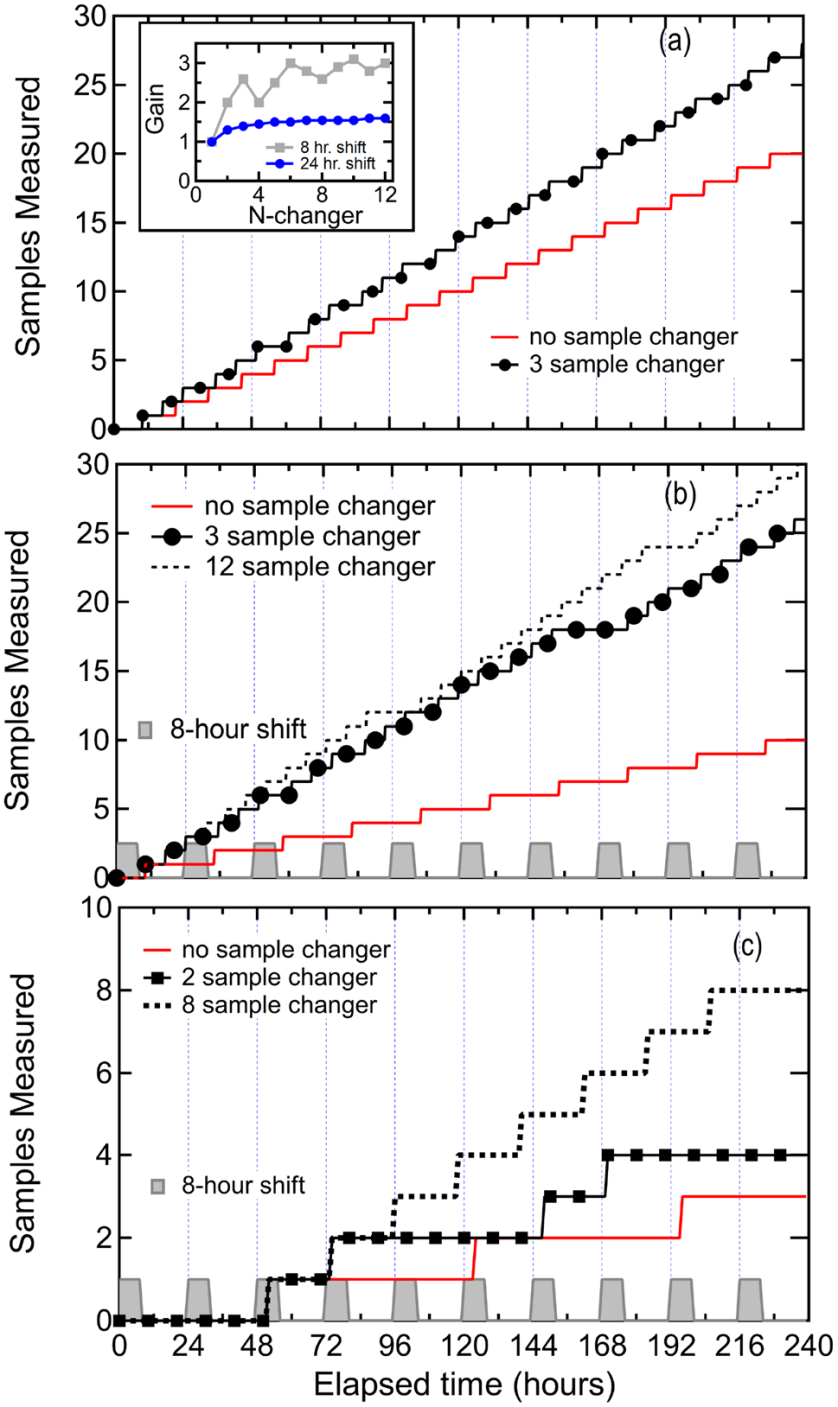
Quantification of sample changer efficiency. (**a**) Number of samples that can be measured as a function of time for operation without (red line) and with a three-sample changer (black dotted line). The label *no sample changer* refers to there being only a single sample available for a measurement cycle. Calculation considers staff being available at all times to change samples. Details of calculation are described in the text. The ordinate of the lines shown increases by one at the completion of each sample measurement. (**b**) Number of samples that can be measured as a function of time for operations without (red line) and with a three (black dotted line) or twelve (black dashed line) sample changer. Calculation considers staff being available to change samples only during a fixed 8 hour shift in each day. Details of calculation are described in the text. The grey shaded area illustrates the portion of time an individual is present to change samples at the instrument. Inset shows the gain factor for a *N*-sample changer when considering 24 hour staffing (blue points) and 8-hour staffing (grey squares). This gain is for the time-intervals chosen for panels (**a**) and (**b**) of the figure and described in the text. (**c**) Samples measured using the 8 hour shift limitations for the ultra-low-temperature sample changer considered as described in the text.

Before examining design considerations of sample changers, we briefly discuss potential gains in efficiency when using such devices to further motivate their use. The measurement process in a CCR sample environment broadly consists of four steps, each which requires a give time $t_n$. Loading a sample into a sample environment, requiring a time $t_1$, cooling the environment to a fixed temperature, requiring a time $t_2$, performing the scattering measurements under different conditions, requiring a time $t_3$, and warming the sample and removing it from the sample environment, requiring a time $t_4$. The process is often repeated multiple times in series during the operational cycle of the neutron scattering facility. Using a sample changer for this process, one would load multiple samples, *N*, into the sample changer, cool them all simultaneously, and measure the samples individually, and then warm up the entire sample changer to repeat the process. Considering fixed measurement times and identical values of $t_1$, $t_2$, and $t_4$ for the sample environment with and without the sample changer option, and considering that someone is available at any time for all sample changes, the sample changer saves the time $(N-1)(t_1 + t_2 + t_4)$ every time it is used.

In Fig. 1, we consider how many samples can be measured in a fixed ten day window of operations. Figure 1a shows the number of samples that can be measured as a function of time for fixed values of $t_1=1$, $t_2=2$, $t_3=7$, and $t_4=1.5$ hours. The red curve shows the number of samples that can be measured with no sample changer and the black dotted curve shows the number of samples that can be measured with a three-sample changer. The time saved allows one to measure additional samples. The inset of Fig. 1a shows the gain in using the sample changer as the ratio of the number of samples measured with the sample changer divided by the number of samples measured without the sample changer in the 10 day time frame. The gain is greater than one and increases as a function of N, but it does not exceed 1.6. This gain is a function of the ratio of the sum of $t_1$, $t_2$, $t_4$ to the value of the measurement time $t_3$. However, a more realistic calculation of efficiency needs to account for finite staffing. Staff are not able to change samples 24 hours a day. Additional time savings are guaranteed if one considers that someone is only available to load and unload the sample changer during fixed amounts of time on a daily basis. To account for finite staffing in Fig. 1(b), we consider the number of samples that can be measured when an individual is present only within an eight hour window or *shift* in a fixed time of day for each day of operations. Now, the gain factor as a function of *N* fluctuates between 1.8 and 3. Further efficiencies could be obtained by building two sample changers. The time $t_1$ would then be performed for the second sample changer while the first sample changer begins its measurement cycle. This strategy also allows the facility to have a spare device ready in case the first device requires repair.

We also consider sample changers built on a dilution refrigerator ultra low-temperature platform. These devices commonly require longer times to change samples due to requirements to evacuate and test sample spaces prior to cooling and the longer times required to reach the lowest temperatures. Figure 1c compares the number of samples that could be measured for single crystals mounted within such a platform using the eight hour shift constraints described earlier. We include the extra time in preparing this sample environment in the time $t_1$ and the extra time in cooling in the time $t_2$ with $t_1=24$ hours and $t_2=6$ hours. We choose a measurement time of $t_3=22$ hours and $t_4=6$ hours for warming the sample environment In ten days, this would allow one to measure three samples. As shown in Fig. 1(c) the 2 and 8 sample changer built on the ultra low-temperature platform allows for 4 and 8 samples to be measured respectively. Automated sample changers have significant potential to increase the throughput of neutron scattering instrumentation across a range of sample environment and measurement suites.

A further practical use of sample changers is the ability they give to screen samples prior to longer measurements. With three different samples loaded, for example, one can perform three short survey measurements to identify which samples will require longer counting times for accurate measurements. This screening capability allows one to quickly prioritize measurements. To accomplish this without a sample changer would require significant effort and time.

## Design considerations

Neutron scattering instruments scatter neutrons into a range of scattering angles measured with respect to the incident beam of neutrons. Sample changers for scattering measurements have geometrical constraints due to the geometry of the neutron detector arrangement. A sample changer needs to accommodate the incident beam and the entire range of scattered beam without obstruction. Two methods have been routinely used to operate a sample changer under these geometric constraints. The first of these is to sequentially place individual samples into the neutron beam while additional samples are held in waiting within a nearby sample reservoir^12,17^. These samples can be held at fixed temperature to reduce the time for cooling, $t_2$. The samples are then translated into the incident neutron beam, either vertically or horizontally, or picked mechanically from a nearby reservoir of samples^24^. The second method is to place samples onto a changer that sequentially places individual samples into the incident neutron beam by rotating about an axis that is orthogonal to the incident neutron beam. This rotation axis can be horizontal or vertical. We will first present design and operational studies of this second type of sample changer. Later in the manuscript we examine the prior described type of sample changer where multiple samples are arranged along a vertical axis and sequentially placed into the beam via a translation motor

There are two sample changer designs that are now used at the thermal chopper spectrometers ARCS^25^ and SEQUOIA^26^ at the SNS at Oak Ridge National Laboratory. Both designs use a rotation concept and have chosen to use three samples. The rotation concept offsets a vertical axis of rotation from the vertical sample center of the instrument and rotates one sample into the beam while the others are blocked by shielding. Such a design makes efficient use of staff time, as described above, and is mechanically simple as it has only one moving part.

**Fig. 2:**
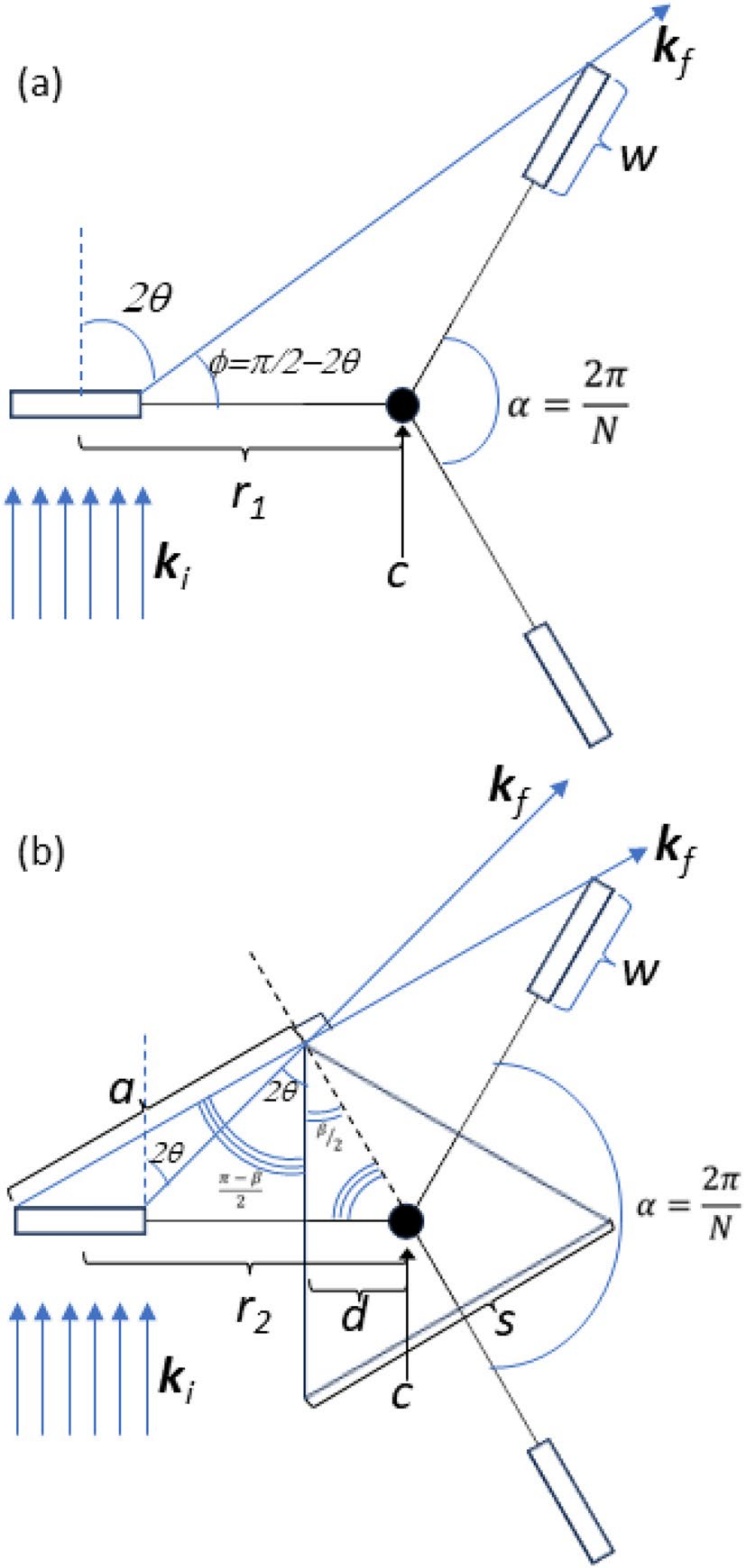
View from above of the scattering geometry for the vertical axis sample changer design described in the manuscript. Geometric parameters are described in the text. (**a**) Three sample changer scattering geometry. (**b**) Shielded three sample changer scattering geometry. Regular triangle centered on the axis of rotation, *c*, represents neutron shielding to prevent scattering between samples that may lead to additional background.

The following discussion describes the design limits of the rotational concept. First we consider ensuring that the samples not in the incident neutron beam do not obscure the currently measured sample’s view of the detector. The ARCS and SEQUOIA spectrometer both have primary detector banks for neutrons scattering to the left of the incident beam; however there is an additional detector bank to the right of the incident beam as viewed from the neutron source. Figure 2a illustrates a schematic of the sample changer design rotating about the axis of rotation *c* offset from the beam center by a distance $r_1$ as viewed from above the scattering plane. The figure depicts a sample of width, *w*, scattering incident wave-vector neutrons, $\textbf{k}_i$, into a scattering angle, $2\theta$, for final wave-vector neutrons, $\textbf{k}_f$. For detectors far from the sample position, the scattering angle for the far left and far right extremes of the sample is approximately the same. There are no obstructions to the neutrons which are scattered to the left of the sample relative to the incident beam. There are potential obstructions for neutrons scattering to the right. The maximum value of scattering angle that does not intersect the additional sample in the downstream position is defined as $2\theta$, as shown in the figure. We also define the angular separation between samples as being $\alpha =\frac{2\pi }{N}$, where *N* is the number of samples that can be loaded onto the changer. We define the angle $\phi = \frac{\pi }{2}-2\theta$ as one of the interior angles of the triangle shown in the figure. From this triangle, one can use the law of sines to determine the value of $r_1$ as a function of *w* and scattering angle $2\theta$:

1 $$\begin{aligned} {r}_{1} = \frac{w}{2}\frac{\cos (2\theta )(1+\cos (\frac{2\pi }{N})) + \sin (2\theta )\sin (\frac{2\pi }{N})}{\cos (2\theta )(1-\cos (\frac{2\pi }{N})) - \sin (2\theta )\sin (\frac{2\pi }{N})}. \end{aligned}$$

**Fig. 3:**
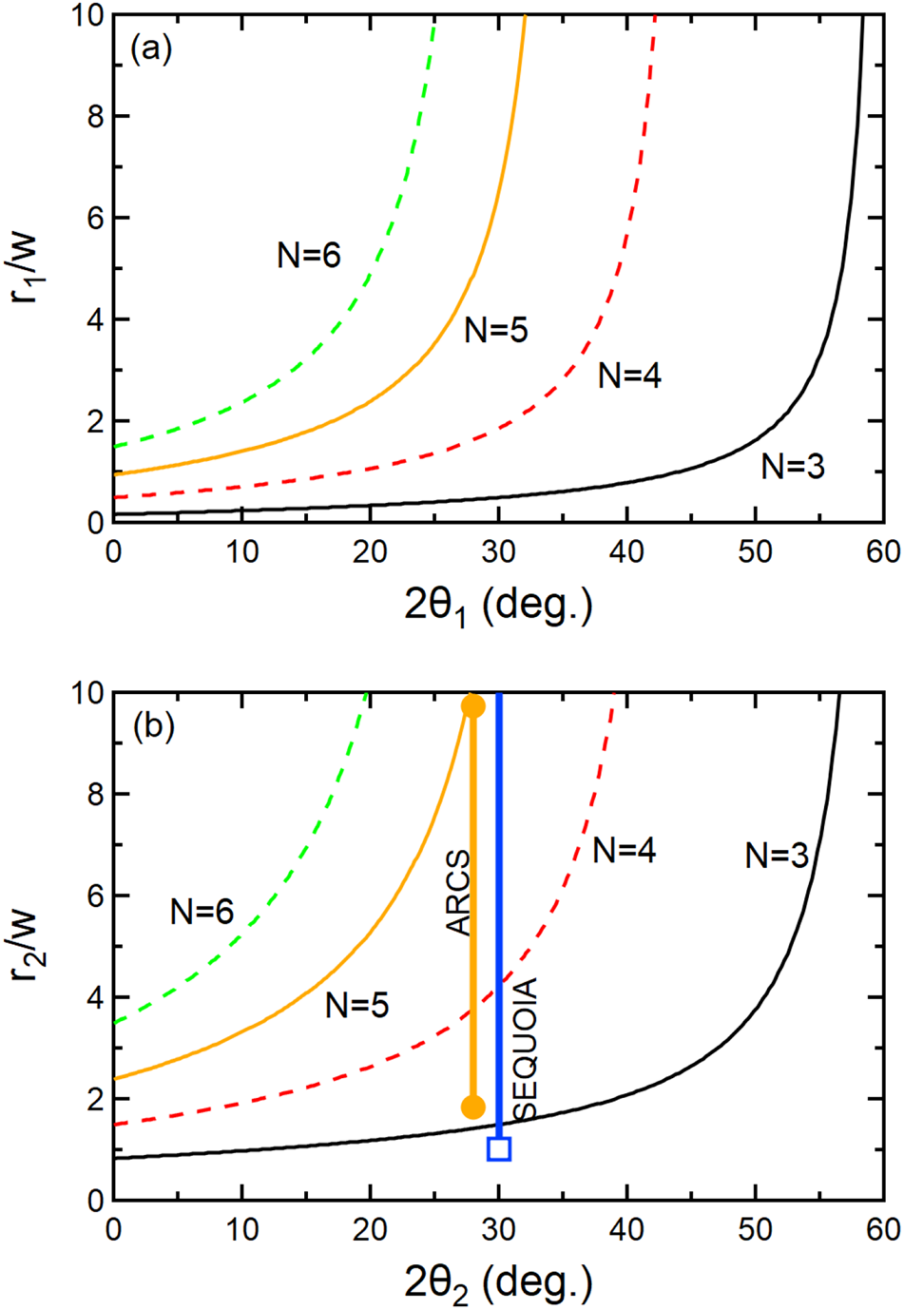
*r*/*w* as a function of scattering angle for different values of *N* sample changers. (**a**) $\frac{r_1}{w}$ as a function of scattering angle for the sample changer geometry shown in Fig. 2a. (**b**) $\frac{r_2}{w}$ as a function of scattering angle for the sample changer geometry shown in Fig. 2b. Vertical lines plotted in (**b**) correspond to the range of sample cans used with the as-built devices for the ARCS and SEQUOIA instruments described in the text.

Figure 3a shows $\frac{r_1}{w}$ as a function of $2\theta$ for several values of *N*. As *N* increases the maximum scattering angle for scattering to the right direction of the sample decreases.

A modification of the geometry shown in Fig. 2a is the inclusion of a piece of *N*-sided shielding in the center position to block the scattered beam from the primary sample from interacting with other samples attached to the sample changer. This is illustrated in Fig. 2b where we define the *N*-sided shielding as a regular polygon with internal angles $\beta$, where $\beta =\pi -\frac{2\pi }{N}=\pi -\alpha$. We also define the length *a* in Fig. 2b such that

2 $$\begin{aligned} \sin (\frac{\pi }{N}) = \frac{a}{r_2+\frac{w}{2}}, \end{aligned}$$

where $r_2$ is the offset of the rotation axis *c* from the beam center. Using the law of sines yields $r_2$ as a function of *w*, the scattering angle $2\theta$, and *N*:

3 $$\begin{aligned} r_2 = w(\frac{\cos (2\theta )}{\sin (\frac{\pi }{N})\sin (\frac{\pi }{N}-2\theta )}-\frac{1}{2}). \end{aligned}$$

Figure 3b shows $\frac{r_2}{w}$ as a function of $2\theta$ for several values of *N*. As more samples are added to the sample changer, there are further restrictions to the maximum scattering angle to the right of the sample. Equation 3 diverges as $2\theta \rightarrow \frac{\pi }{N}$ which means for a given number of samples there is a limit to the scattering angle that can be used that is independent on the sample width or radius of the device. This result may be important in addition to factors like access to the sample area and total scattering angle coverage for direct geometry spectrometers that are currently under development like CSPEC^27^ or CHESS^28^. The geometry in Fig. 2b can be used to determine the distance from the center of rotation to the edge of the shielding polygon, *d*:

4 $$\begin{aligned} d = (r_2 + \frac{w}{2})\cos ^2(\frac{\pi }{N}). \end{aligned}$$

The length of the sides of the shielding polygon,*s*, are also a function of the sample width and radius:

5 $$\begin{aligned} s = 2(r_2 + \frac{w}{2})\cos (\frac{\pi }{N})\sin (\frac{\pi }{N}). \end{aligned}$$

Operationally the 3 sample changer is usually filled with two samples and an empty can. For reducing direct geometry spectroscopy data, typical practice is to measure an empty sample container, under the same conditions as the sample, and then subtract the two measurements. When running without a sample changer, the usual mode is to save the empty can measurements until the end of the experimental time available. With the empty-can as one of the samples in the changer, the users can interleave their sample and empty measurements and thus complete a background subtracted measurement during their allotted measurement time as well as more finely tune the counting times of both the sample and the empty can.

The SEQUOIA sample changer is based on a bottom loading CCR with a 3-sample rotation design that can be quickly converted to a single crystal mode of operation with a single sample at the center position. The ARCS sample changer is based upon a top-loading CCR platform and includes a 3-sample changer that is able to be heated to $T=700$ K. Both platforms use the shielding configuration shown in Fig. 2b to help reduce background scattering.

## The SEQUOIA 3-sample changer

The initial design of the SEQUOIA 3-sample changer was born out of the need to mitigate experimental demands on staff due to the high flux of neutrons at the SEQUOIA beamline. Even when the source was operating at 800 kW it was quickly determined that 4 hours was sufficient for a powder measurement, and, after multiple back-to-back experiments of changing samples every four hours, a method was needed to operate the instrument for 24 hours between staff intervention. The 3 sample changer concept met this criteria. We also had experience operating a CCR with a rotation stage for single crystal studies. By simply offsetting the rotation from the center position used for single crystals, we could use the same device and quickly implement a sample changer on SEQUOIA that would realize the gains shown in Fig. 1. The success of this device led to further optimization and refinement. The refinement process, and the current device that is in regular use, is described here. An additional requirement is to be able to change quickly between the single crystal and sample changer configurations of the device. The device also needed to be able to have samples, thermometers, heaters and neutron shielding all placed at standardized positions for consistent and reproducible measurements.

**Fig. 4:**
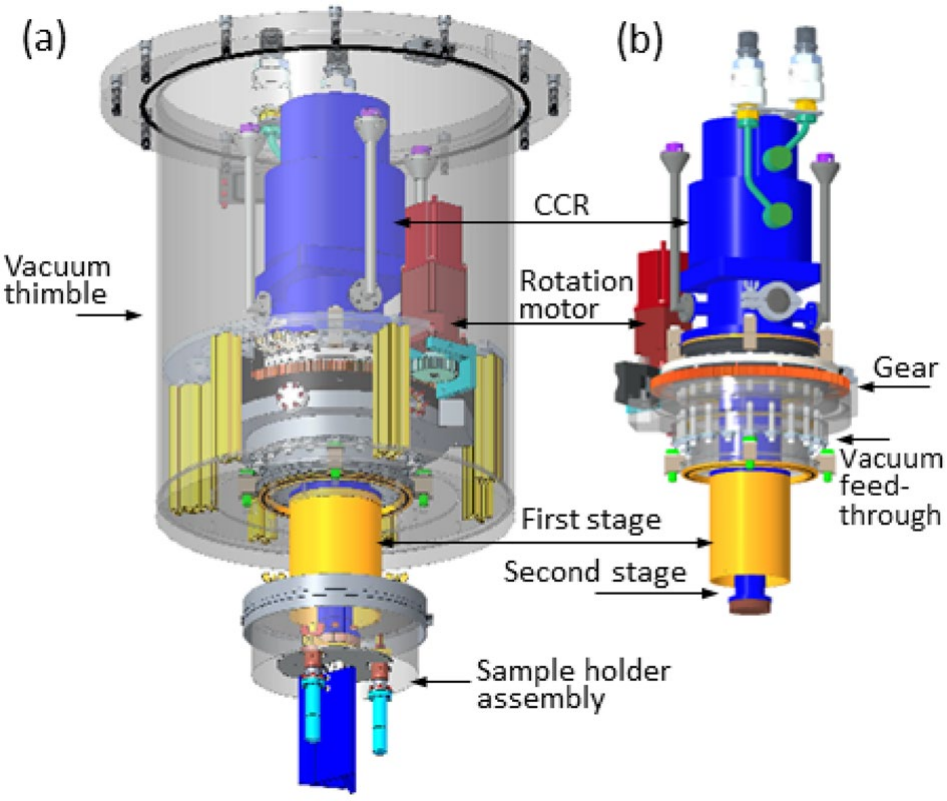
The SEQUOIA bottom loading 3-sample changer. (**a**) View of the entire apparatus including the vacuum thimble, shown as transparent grey in the image. The CCR is a *ARS DE-210 sf* model. The first and second stage of the CCR are within the vacuum space of the instrument. The sample holder assembly hangs from the end of the second stage of the CCR. (**b**) Detailed view of the CCR cold-head and feed through rotation stage. A computer controlled stepper motor turns a gear that provides rotational motion about the vertical axis of the device.

The SEQUOIA 3-sample changer is mounted upon an *ARS DE-210 sf* bottom loading CCR platform. Figure 4 is a rendering of the entire SEQUOIA 3-sample changer apparatus. The CCR sits within a stainless steel thimble that provides a rigid vacuum boundary for the SEQUOIA instrument^26^. The thimble has 6.35 mm thick sidewalls and a 25.4 mm thick base. This thimble is loaded into the sample vacuum space of the SEQUOIA instrument from above via a crane. Because the beam position at SEQUOIA is 749 mm below the bottom of the standard vacuum flange, the thimble configuration allows the cold-finger of the CCR to be as short as possible in order to provide as much cooling power to the sample as possible. The CCR is mounted to a rotation stage through the vacuum boundary as shown in Fig. 4b. A computer controlled stepper motor drives a gear that in turn meshes with a gear on the outside of the vacuum feed-through to provide rotation about the vertical axis of the device. This rotation is used to access different scattering wave-vectors for single crystal samples, or to switch between powder samples in the 3-sample configuration. The backlash in the gear rotation is reduced via software by always making a counter clockwise rotation as the last step in the motion.

**Fig. 5:**
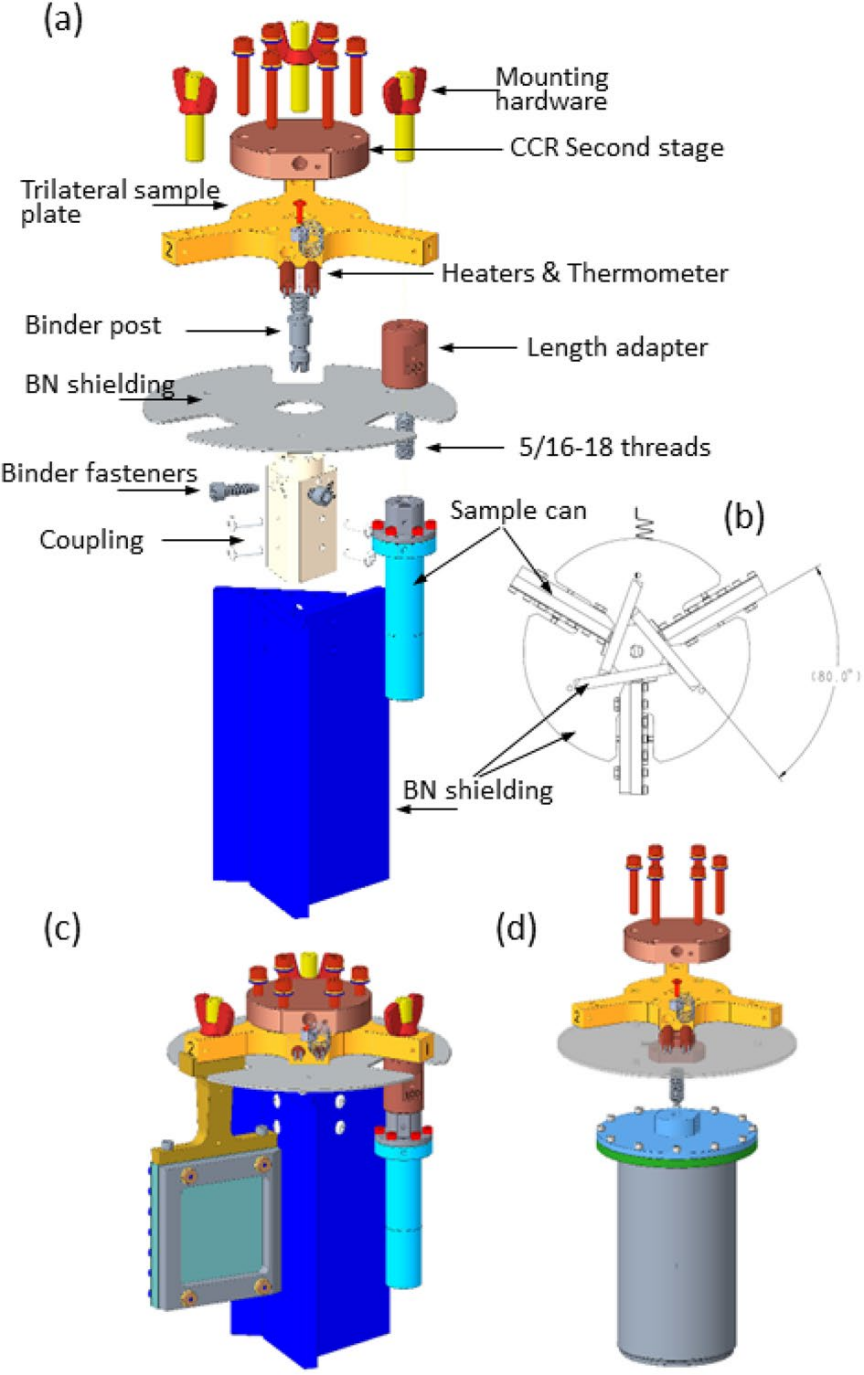
Details of the SEQUOIA bottom loading 3-sample changer assembly. (**a**) Exploded view of the sample changer assembly. (**b**) Bottom view detailing the arrangement of the boron nitride baffles. (**c**) View of the assembled 3-sample changer with both cylindrical (right) and flat plate (left) sample cans attached. (**d**) Exploded view of the assembly showing how single crystal samples are attached to the center of the trilaterial sample plate.

Figure 5 provides additional details regarding the sample holder assembly for the SEQUOIA bottom loading 3-sample changer. Figure 5a shows an exploded view of the 3-sample changer assembly. The value of $r_2$ for the sample changer is 50.8 mm. A trilateral sample plate made of oxygen free high conductivity copper is fastened to the end of the CCR’s second stage using brass machine screws. The trilateral plate has clear holes at the end of each arm and a 5/16-18 thread per inch hole in the center position. Two heaters are mounted within the trilateral plate in circular clear holes with a threaded fastener that can retain the heaters in position. One thermometer is located on the trilateral plate, and three additional thermometers (*Lake Shore Cryogenics* *DT 670 *silicon diode sensors in the *CU* package) are available to mount to individual samples or other locations on the assembly. A steel binder post is threaded into the central threaded holes of the trilateral plate. This binder post has a smooth outer surface with a notch that allows the binder fasteners to suspend the coupling and boron nitride neutron shielding between the sample cans. The coupling shown in Fig. 5a is an aluminum triangular prism that suspends from the binder post using three steel binder fasteners. The binder fasteners are machine screws with their tips smoothed so that the fastener can be threaded into the coupling and support the coupling on the binder post using the smoothed portion of the fastener. The coupling has a fixed pin on its top surface that fits into a single clear hole in the trilateral sample plate. This pin provides reproducible positioning of the sample changer assembly. The coupling also holds an  3 mm thick disk of boron nitride in place above the samples. The coupling also is used to fasten boron nitride shielding baffles in place between the samples. These shielding baffles are machined from  6 mm thick boron nitride. Figure 5b is a bottom view of the boron nitride shielding between the sample cans. The shielding above the sample position prevents neutrons that are scattered upwards from the samples to also scatter off of the CCR hardware and subsequently contribute additional background to the measurement. The shielding between samples prevents multiple scattering events between samples from occurring.

Figure 5c shows the assembled view of the SEQUOIA 3-sample changer. Brass 5/16-18 threads per inch threaded posts and thumbscrews are used to hold samples in position on the trilateral sample plate. Flat plate and cylindrical powder cans can be used interchangeably on the 3-sample changer as shown in Fig. 5c. Flat plate cans have custom masking made of neutron absorbing material in place on the incident beam side of the sample can^29^. Custom cut cadmium shielding is used for shielding around the oxygen free high conductivity copper parts that join the sample cans to the trilateral sample plate. The flat plate sample cans used at SEQUOIA have a maximum sample width *w* of 50 mm. Figure 3b shows a vertical line corresponding to the ratio of $\frac{r_2}{w}$ for the range of sample can sizes used at SEQUOIA. We consider a 3 mm diameter sample can as the likely narrowest can that would be used. An aluminum foil heat shield is placed around the entire assembly and fixed to the first cooling stage of the CCR. This heat shield is not shown in the figures.

For single crystal measurements, the central 3-sample changer assembly is removed. This is done by loosening and removing the three binder fasteners and sliding the coupling with its attached boron shielding down from the binder post. The binder post is unscrewed using a flat head screwdriver. The central threaded hole of the cold-finger mounting plate is then used to suspend single crystal samples into the neutron beam. An ultrathin single crystal sample holder^30^ is shown as part of an exploded view of the assembly in Fig. 5d in the single crystal configuration of the device. Oxygen free high conductivity copper parts of fixed length are used to account for height differences that may be present in single crystal sample mounts. These adapters are also used in case powder cans are not completely full and need to be suspended higher in the incident neutron beam. A boron nitride shielding disk is held in place on a ledge of the copper spacer to shield neutrons from scattering up into the CCR. An angular notch is made in this disk to allow for thermometry wires to access single crystal samples for monitoring temperature without providing a line-of-sight for scattered neutrons to contribute to additional background to the measurement.

**Fig. 6:**
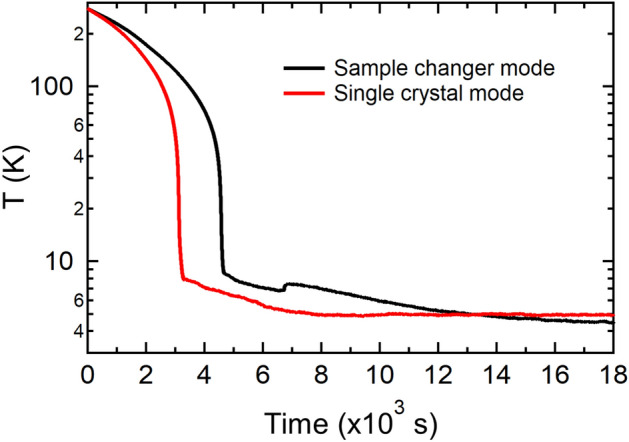
Cooling test of the SEQUOIA sample changer as a function of time. Data are thermometer measurements with the thermometer located on the CCR cold-head. Black curve is for the CCR in the three sample changer configuration. The red curve is for the CCR in the single crystal mode of operation.

The SEQUOIA 3-sample changer is used regularly in operations of the instrument. The additional mass from the entire sample changer assembly on the second stage of the CCR does increase cool-down time of the device. Cool down times to base temperature of $T\approx 5$ K are approximately 1.5 hours. We provide here measurements of the cooling and heating curves of the SEQUOIA 3-sample changer. Figure 6 shows the cooling curves for the SEQUOIA 3-sample changer in both the single crystal and the sample changer configurations. The sample changer configuration included the additional mass of shielding and three flat plate sample cans attached to the changer. Approximately one hour is required to have the sample environment reach below $T=8$ K in the single crystal configuration. An additional thirty minutes is required for the 3-sample changer configuration to reach temperatures below $T=8$ K. While there is some thermal lag in cooling due to the extra mass of the sample changer, this additional time is offset by the time saved in using the sample changer for the experiments.

**Fig. 7:**
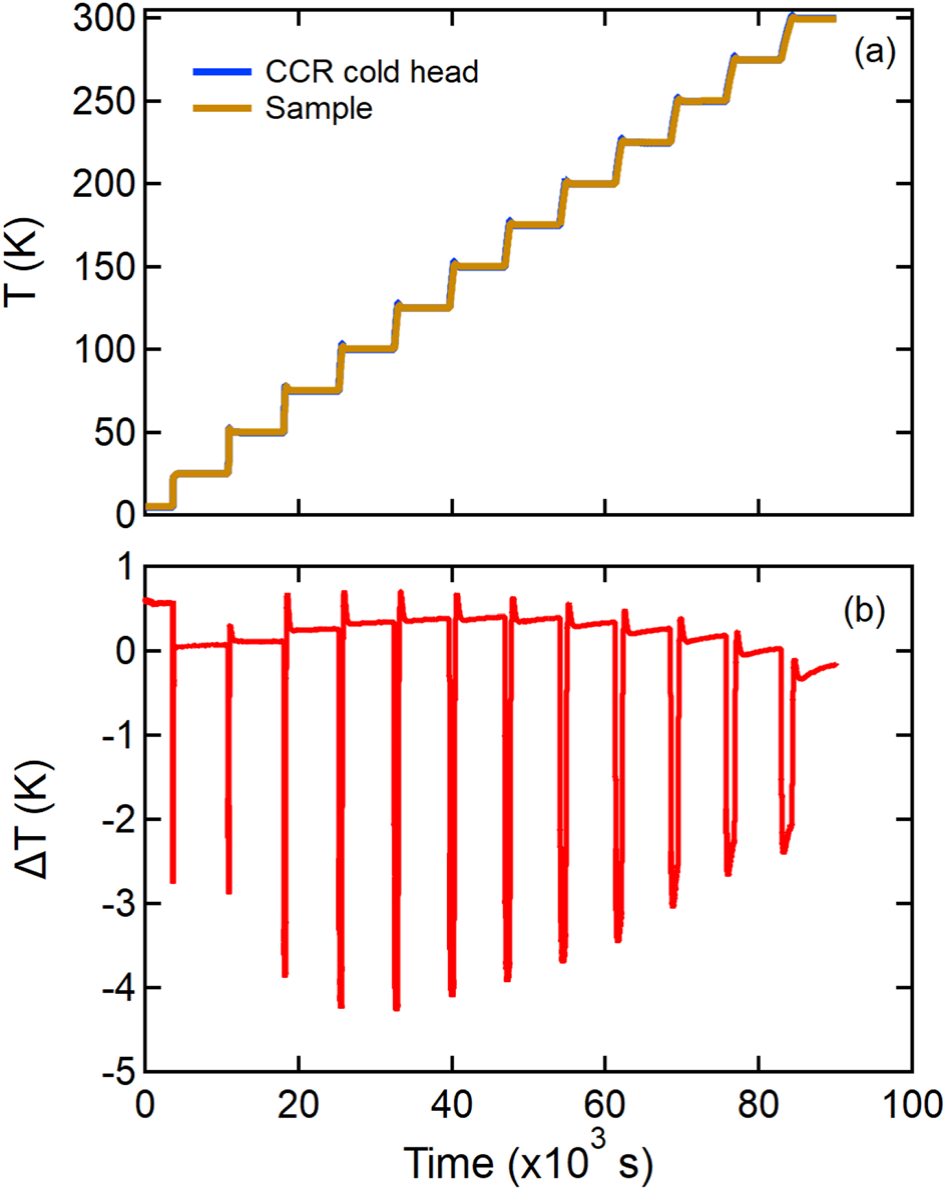
Temperature test of the SEQUOIA 3-sample changer. (**a**) The temperature of two different sensors on the bottom loading CCR. The Heater Block sensor is placed close to the heater and is used for control. The Sample sensor is attached to the sample can (**b**) The difference between these two thermometers.

Figure 7a shows the temperature on the cold head and the temperature on one of the three samples for a series of heating steps with the CCR measured in the 3-sample changer configuration. Both temperatures track one another very closely. Figure 7b is a plot of the difference in the curves shown in Fig. 7a. The offset in temperature during a temperature change is approximately 4 K. As thermal equilibrium is approached, this difference in temperature diminishes to less than one Kelvin.

**Fig. 8:**
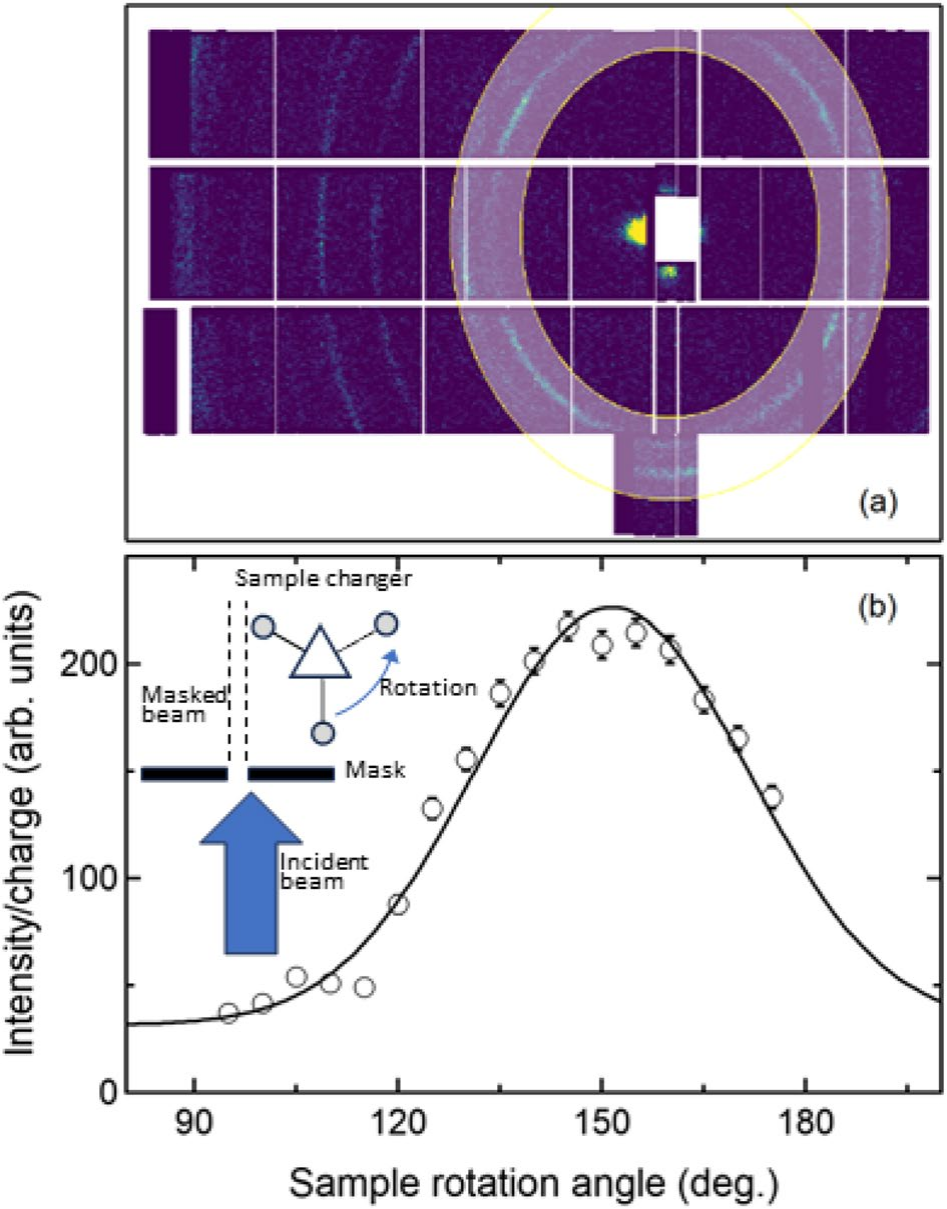
Method to determine sample position on the SEQUOIA 3-sample changer. (**a**) SEQUOIA detector array illustrating detector counts as a function of position on the array. The detector array is viewed from the sample position of the instrument. The white box in the center of the figure is the beam stop of the instrument. The lighter shaded annular region corresponds to a region of interest set to integrate the detected neutrons for the first two Bragg peaks of an empty aluminum sample can. The bright spot to the left of the beam stop is due to masking a significant portion of the right side of the incident beam on the sample as described in the text. (**b**) Integrated scattering intensity normalized to proton charge as a function of sample rotation angle and integrated over the region of interest shown in panel (**a**). Inset depicts the geometry of incident beam and sample rotation described in the text to determine the sample positions for the SEQUOIA 3-sample changer.

We have refined a simple technique to accurately locate sample positions for the SEQUOIA 3-sample changer. This is depicted in the inset of Fig. 8b. We first narrow the right portion of the incident beam at or just beyond the midpoint of the incident beam using the SEQUOIA motorized in-vacuum slits. We then sweep the sample through the masked beam using the rotation motor. The maximum neutron count rate on the instrument detector then corresponds to the rotation angle at which the sample is aligned in the incident beam. Figure 8a shows a view of the detector array at SEQUOIA as viewed from the sample position for one of these rotation scans for an empty aluminum sample can. Note the bright spot to the left of the beam stop location. This asymmetrical beam divergence is due to the right mask being closed beyond the midpoint of the incident beam. The detector array shows a series of aluminum Bragg peaks. We have highlighted a region of interest for the two lowest angle nuclear Bragg peaks for aluminum using an annular region of interest. The detected neutrons in this region of interest are integrated as a function of sample rotation angle. This value is normalized to the proton charge on the target at the source and plotted as a function of rotation angle in Fig. 8b. We fit this curve to a Gaussian shape with a constant background and set the centroid of the Gaussian to be the position when the sample is centered in the incident beam. These analysis steps are performed using the Mantid software^31^. Scans of the mask positions are performed to set the mask to match the incident beam to the width of the sample can and the height of the sample that is located within the can. This is accomplished with additional scans of the top, bottom, left and right mask motors and determining when the neutron counts on the detector begin to be diminished from masking the neutrons incident upon the sample.

### Vacuum flanges for the SEQUOIA 3-sample changer

**Fig. 9:**
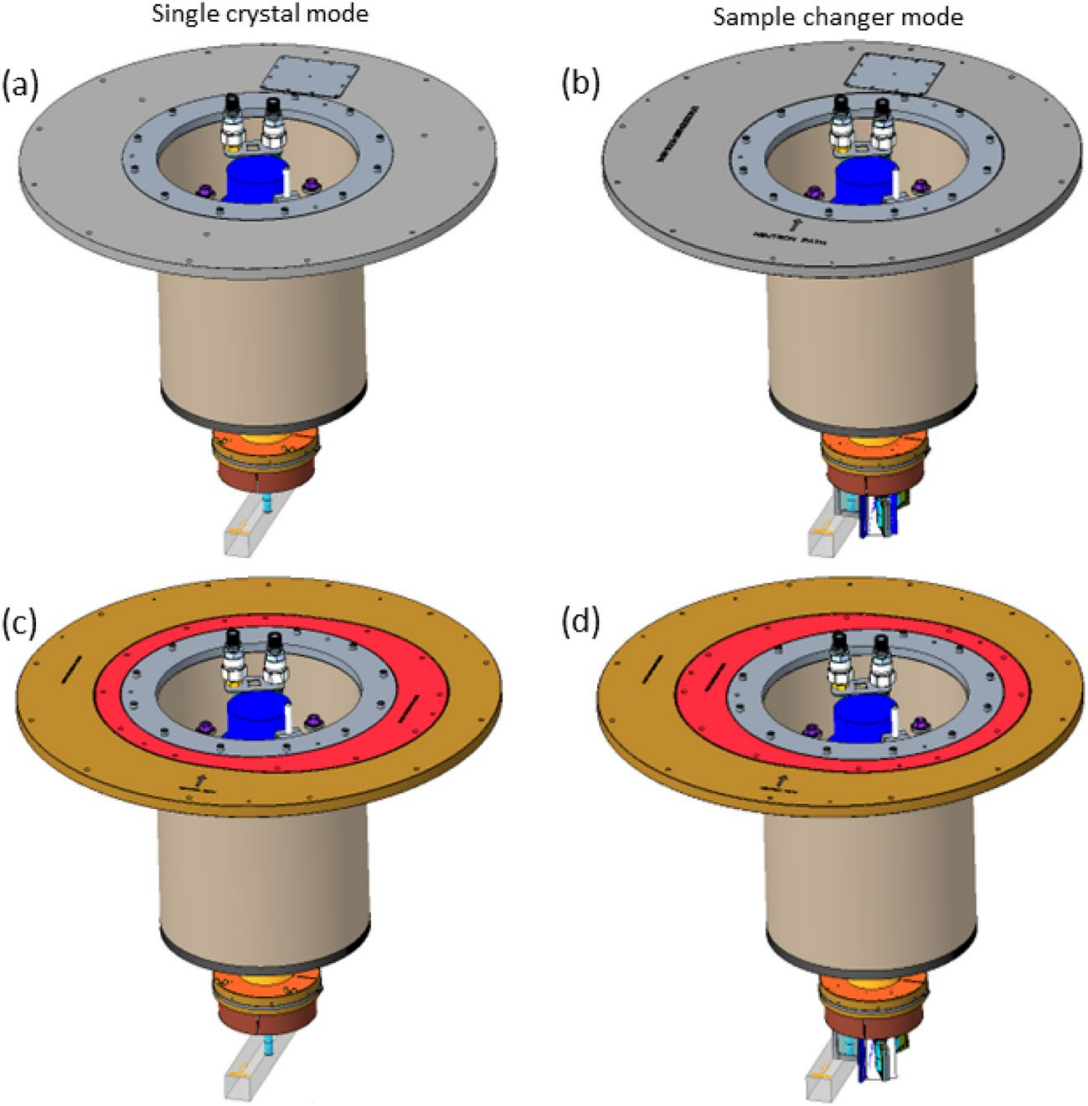
Flange configurations available for the SEQUOIA 3-sample changer. In each of the flanges shown, the rectangular prism at the bottom of the sample environment represents the square profile of the incident neutron beam at the SEQUOIA instrument. The large flanges in each configuration has the profile of the 812.8 mm standard flange of the facility. (**a**) Original flange configuration of the SEQUOIA 3-sample changer mounted in the single crystal configuration. (**b**) Original flange configuration of the SEQUOIA 3-sample changer mounted in the offset position for sample changer mode of operation. (**c**) Revised flange of the SEQUOIA 3-sample changer mounted in its single crystal configuration. (**d**) Revised flange of the SEQUOIA 3-sample changer mounted in its offset position for sample changer mode of operation. The brown flange in the revised version (**c**) and (**d**) remains fixed in position for changing between the single crystal and sample changer modes of operation.

The standard vacuum flanges for flange mounted sample environments at the Spallation Neutron Source allow sample environments to suspend into the neutron beam while preserving vacuum of the instrument. They are nested with diameters of 406.4, 812.8, and 863.6 mm with openings centered on the instrument’s vertical axis passing through the sample position. The thimble of the SEQUOIA CCR has a 406.4 mm standard flange diameter and is shown mounted within the 812.8 mm standard flange in Fig. 9a in the single crystal configuration. An offset hole was made in a 812.8 mm standard diameter flange to accommodate the SEQUOIA 3-sample changer CCR in the sample changer configuration. This is shown in Fig. 9b. The weight of the SEQUOIA CCR assembly with its thimble is approximately 90.7 Kg. The weight of the large flanges in Fig. 9a,b are 156 Kg and 160.6 Kg, respectively.

To change between the original flanges shown in Fig. 9a,b required six uses of the overhead crane with a total weight lifted of approximately 816.5 Kg. A new flange was designed to reduce the number of lifts. This flange is shown in Fig. 9c,d for the single crystal and sample changer modes respectively. This new flange consists of a nested asymmetric vacuum flange, shown as red in Fig. 9c,d, that can be quickly rotated to switch between modes. This red flange weighs 49.4 Kg. The brown flange shown in Fig. 9c,d stays fixed in position for this process. This new flange now only requires three lifts with the instrument crane to change configurations with a total weight lifted of approximately 231.3 Kg

## The ARCS 3-sample changer

Often at neutron spectrometers, top loading sample environments are used. These sample environments remain installed in position, and sample sticks or inserts are placed within them from above the environment to change between samples. Typically these sticks hold a single sample. Two common *top-loaders* are the so-called *Orange Cryostat* and a top loading CCR both of these devices come in multiple sizes. The large (100 mm) bore configuration is the one used here. For certain beam and instrument geometries, this is enough space to place a sample changer inside the bore diameter. Both of these devices also have a rotation stage that can be used with a single sample stick for single crystal studies. Therefore the motion is encoded and programatically controlled for backlash just as the SEQUOIA bottom loading CCR. With the existing rotation, and the inspiration of the successful SEQUOIA design, we chose to start by adding the necessary offset flange and 3 sample sticks for the top loading CCR. A continued program that will enable use down to 2K is envisioned by making similar sticks for the *Orange Cryostat*.

In order to fit in the 100 mm diameter sample space we chose to use only cylindrical powder cans with a diameter up to 15.9 mm. The bottom point for the line marked ARCS in Fig. 3 shows that for this can size the neighboring cans see no scatter from the one in the beam. One also notices that this line crosses the N=4 line before the minimum can size of 3mm, showing a 4 can design is feasible for smaller samples. Restricting the design to smaller diameter sample cans than those used in the bottom loading design means that only a single heater and thermometer are required for temperature control. The temperature limits of this top loading design are controlled by the temperature limits of the CCR. For the case here the base temperature is 5 K and the upper limit is 700 K. For operation below 300 K, the VTI of the CCR is filled with a partial pressure of He exchange gas. For operation above 300 K, vacuum is used in the VTI. The CCR is equipped with a remote controlled exchange gas control system that can be operated from the data acquisition system, thus allowing for straightforward transitions between the $T<300$ K and $T>300$ K configurations.

**Fig. 10:**
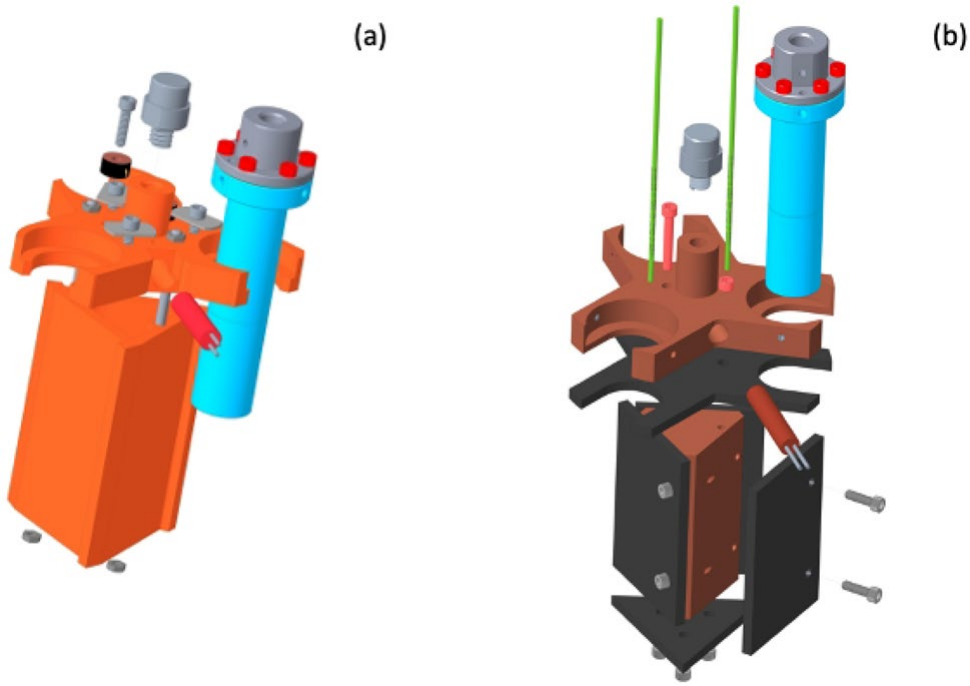
Model views of the ARCS sample sticks. Panel (**a**) shows the sample portion of the cold stick. A single sample can is shown in light blue. The orange pieces were made out of 3D- printed $\mathrm {B_4C}$ and Al. The orange piece holds the samples and there are 3 grey aluminum screw down clips to ensure the sample has good thermal contact. The center grey piece shows the attachment to the stick. The black and Cu colored component is the Si-diode thermometer and the red cylinder is the heater that is inserted into the sample holding piece. Panel (**b**) shows the sample portion of the Hot stick. The Cu colored pieces provide support to hot pressed B4C (black pieces that provide the shielding. The green wires represent the thermocouples. The heater, sample can, and stick mount are the same as for the cold stick.

Though, in principle, one could access the entire temperature range of the cryostat, in practice other design features, e.g. material choice, thermometer choice, come into play so there are two different 3-sample sticks: one for the lowest temperature operation with silicon diode thermometers (Cold Stick) like the bottom loading design and the other with thermocouples for highest temperature operation (Hot Stick). Views of the design models for the sample end of both sticks are shown in Fig. 10. Both designs have the same dimensions; a radius of 29.2 mm and 8 mm clearance to the VTI wall. The Hot Stick, shown in Fig. 10b is limited to above 30 K due to the inaccuracy of the thermocouples at lower temperature; however, this stick can be operated to the maximum temperature of the CCR. For the Cold Stick, shown in Fig. 10a the base temperature of the cryostat is $\sim 5$ K and has a high temperature limit of 400 K, primarily due to indium o-rings in the device. For the low temperature stick, the majority of the mount was additively manufactured from Boron Al material^29^. This provides thermal conductivity and absorbs scattered neutrons. It is two pieces. One is the triangle for blocking the scattered neutrons from other samples, the other holds the samples. These two pieces are clamped together with two screws through the body of the triangle shielding. The cans are clamped down with an Al clip, shown in gray. Thus providing a large surface for thermal contact to the mount. Finally an Al mount is provided to connect the assembly to the sample stick. The Black piece is the Si Diode thermometer used for temperature control and the Red piece inserted from the side is a 50W resistive heater. Temperature is monitored and controlled from these two devices using a Lakeshore temperature controller that also controls a heater where the CCR joins the VTI. The VTI is filled with He exchange gas to facilitate thermal stability and temperature control.

For the Hot stick the mount and a mount for the neutron shielding were manufactured of oxygen free high thermal conductivity (OFHC) copper. Hot pressed $\mathrm {B_4C}$ is used for the absorbing material that covers the neutron facing sides of the Cu and is attached with screws. We did not use the additively manufactured $\mathrm {B_4C}$ Al parts as most of this material is Al and may soften at the highest temperatures. The green lines indicate the thermocouples and a 50W heater is indicated by the red cylinder that is slipped in the heater block. The sample holder portion is attached to the stick with the grey Al piece. Set screws are used from the side to keep the samples in place.

Note that changeout time efficiencies could be improved by making an additional stick that can be “on deck” ready for when the samples in the beam are complete. Furthermore, both of these sticks have a manual vertical adjustment of $\pm 25$ mm to accommodate partially full cans. This capability could be combined with the smaller sample height configurations, described in the next section, to allow for a six sample configuration.

**Fig. 11:**
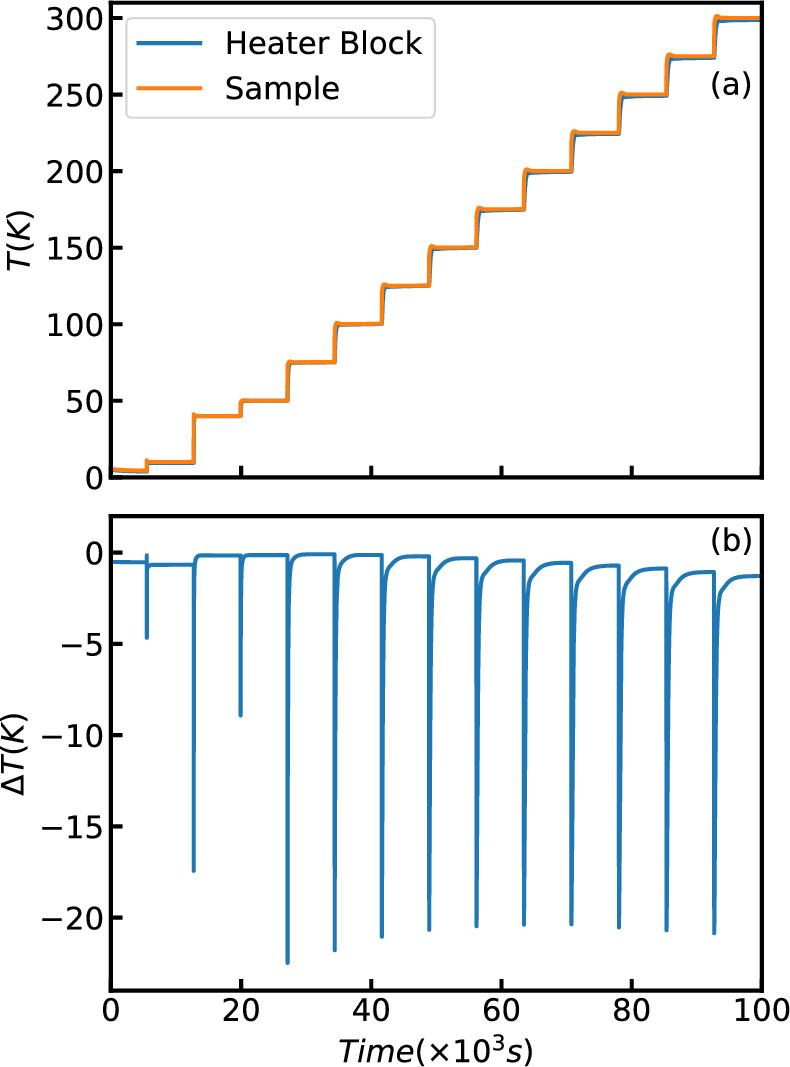
Temperature test of the cold stick. (**a**) The temperature of two different sensors on the cold stick. The Heater Block sensor is placed close to the heater and is used for control. The Sample sensor is in the sample can in a Si powder (**b**) The difference between these two thermometers.

Each stick was tested to see how long the sample takes to come to equilibrium. To model a typical sample 11 gm of Si powder (Alfa Aesar cas#7440-21-3) we placed in a 60 mm tall Al can with a diameter of 16 mm. A thermometer was buried in the powder at the sample center. For the cold stick 14 different temperatures were measured. One was at the base temperature of the cryostat and the others were between 10 K and 300K. Two thermometers were used: one on the heater block and another encased in a Si powder in a sample can. The readings of the two thermometers are shown in Fig. 11a and the difference between the these values is shown in Fig. 11b. For all temperatures above 10K with this stick the the two thermometers are less than 0.5% apart.

**Fig. 12:**
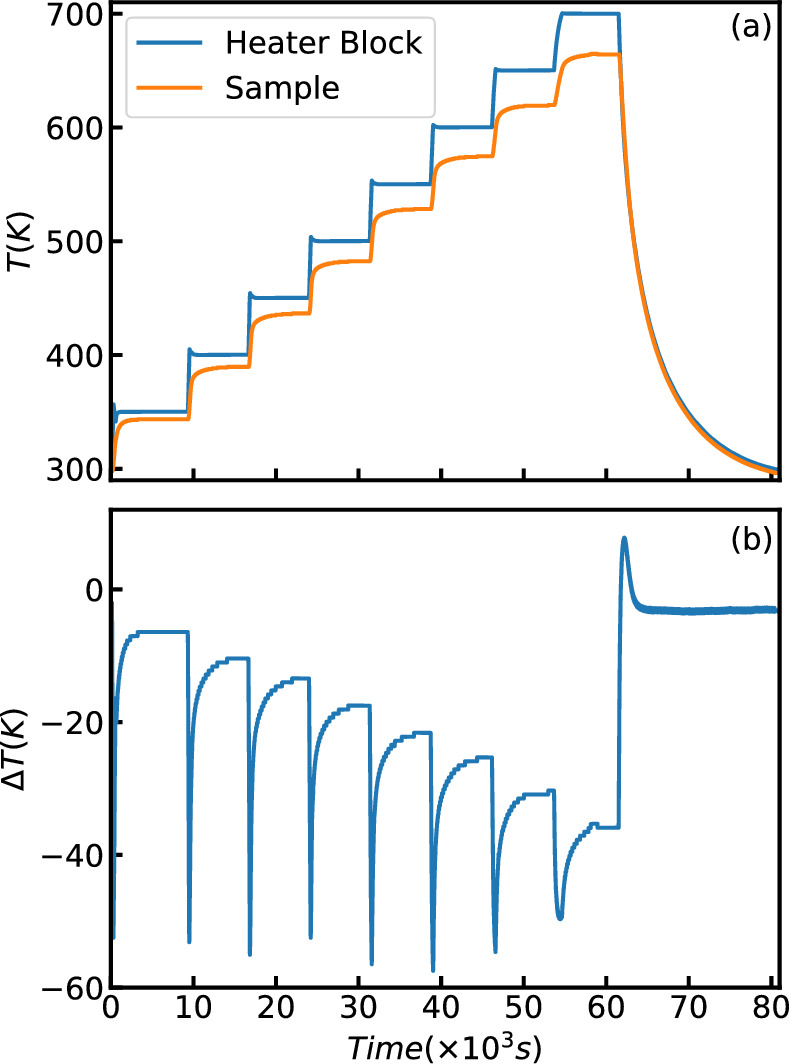
Temperature test of the Hot stick. (**a**) The temperature of two different sensors on the Hot stick. The Heater Block sensor is placed closed to the heater and is used for control. The Sample Sensor is in the sample can that is filled with Si powder. (**b**) The difference between these two sensors shows the time to equilibrium and the T offset for the Sample sensor.

For the hot stick 8 temperatures were measured using a set point from 300K to 700K. A thermometer close to the heater and one in the sample can were monitored. Each temperature was measured until the system reached equilibrium. The temperature curves are shown in Fig. 12a. The difference between these two thermometers is shown in Figure 12. When the sample chamber of the cryostat is under vacuum, the thermal contact between the heater and the sample is not as strong thus the temperature difference gets larger as the temperature increases. Nevertheless, upon reaching equilibrium, the thermometers are within 5% of each other.

## Vertically stacked samples

Earlier we described a type of sample changer that works via a single translation through the beam for a series of samples. Here we describe implementation of this type for another spectrometer at the SNS. At the Cold Chopper Neutron Spectrometer (CNCS) of the SNS^32^, it is common to use ultra low-temperature (ULT) devices (T < 1 K) in order to access lower temperature portions of materials’ phase diagrams. For these sample environments, sample changes require more time than for standard cryostats and CCRs. For $^3$He refrigerators (base temperature $T\approx 250$ mK) and dilution refrigerators (base temperature $T\approx 50$ mK), typical sample change times including a return to base temperature are six and fourteen hours, respectively. In addition to this elapsed time, the sample changes require the support of specialized technical staff. These factors motivated the development of vertically stacked sample holders, which allow for changing between samples within the ULT device to be essentially instantaneous. There are two general classes of samples, single crystals and powders, for which separate sample holders were deigned and fabricated.

The CNCS has a vertical elevator that allows one to translate the sample environment up to 200 mm without interfering with the nearby equipment. A constraint for ULT devices is the small size of the cold space in the cryostats. The beam size at CNCS may be changed by adjusting the last section of neutron guide on the instrument resulting in a typical beam size that is 1 cm wide by 1.5 cm tall.

**Fig. 13:**
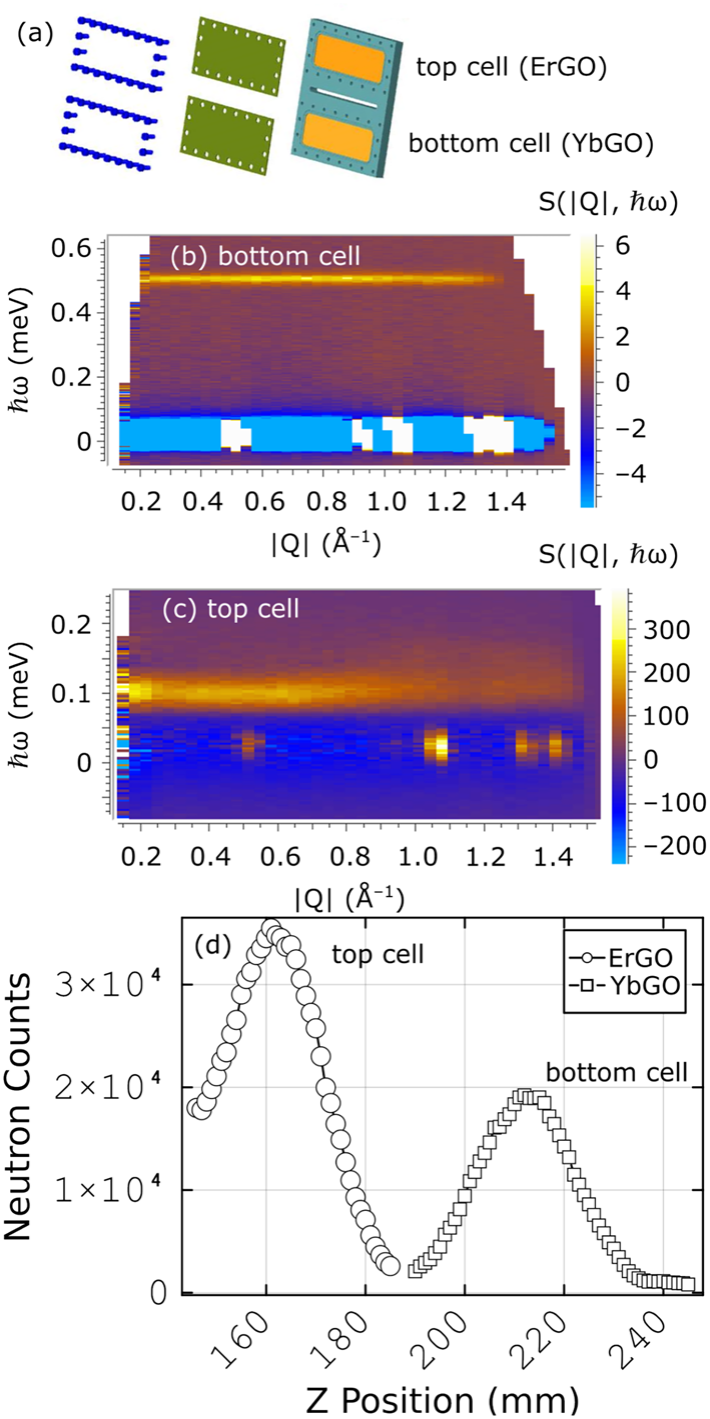
(**a**) Schematic of a double cell vertical sample holder for usage with ULT devices. Yellow portions indicate the two regions where samples are respectively placed within the cell. The cell is sealed with a thin plate of aluminum using an indium seal with an atmosphere of helium gas. This hermetic seal encourages the powder to be at thermal equilibrium with the cryostat sample environment. (**b**) Inelastic scattering spectrum from the bottom cell shown in panel (**a**). (**c**) Inelastic scattering spectrum from the top cell shown in panel (**a**). (**d**) Integrated elastic neutron counts as a function of cell translation position while mounted at the CNCS beamline.

These efforts began with a two-sample flat plate copper cell for powders as shown in Fig. 13a. The body of the cell is made from copper, while the faces are thin aluminum to minimize background. The spacing between the centers of the two samples is 50 mm, and centering is done with the neutron beam using a signal from the sample itself in the detector array (e.g. a Bragg peak). The prototype experiment for this cell was on two pyrogermanate samples, which was subsequently published^33^. A vertical scan, on a Bragg peak of each sample, shows the centering procedure, Fig. 13d. There is no cross-contamination between the samples in the detector array, as seen in the vertical scan and in the full spectra measured for the two sample positions, Figs. 13b,c.

The success of this initial effort led to additional developments for powder vertical sample holders. Specifically, an array of cylindrical aluminum sample holders appropriate for $^3$He inserts and for standard cryostat stick holders. These developments include monolithic and modular geometries, as well as the capability to have annular inserts for cylindrical geometries that require thin samples. CNCS also currently has a three sample changer for its liquid helium, i.e. orange, cryostat that is built in the style of the ARCS sample changer described above.^34^ The cylindrical sample can designs are compatible with this three-sample rotational sample changer, if one includes the ability to vertically translate the sample environment at CNCS, this allows for up to six samples to be mounted in the orange cryostat.

**Fig. 14:**
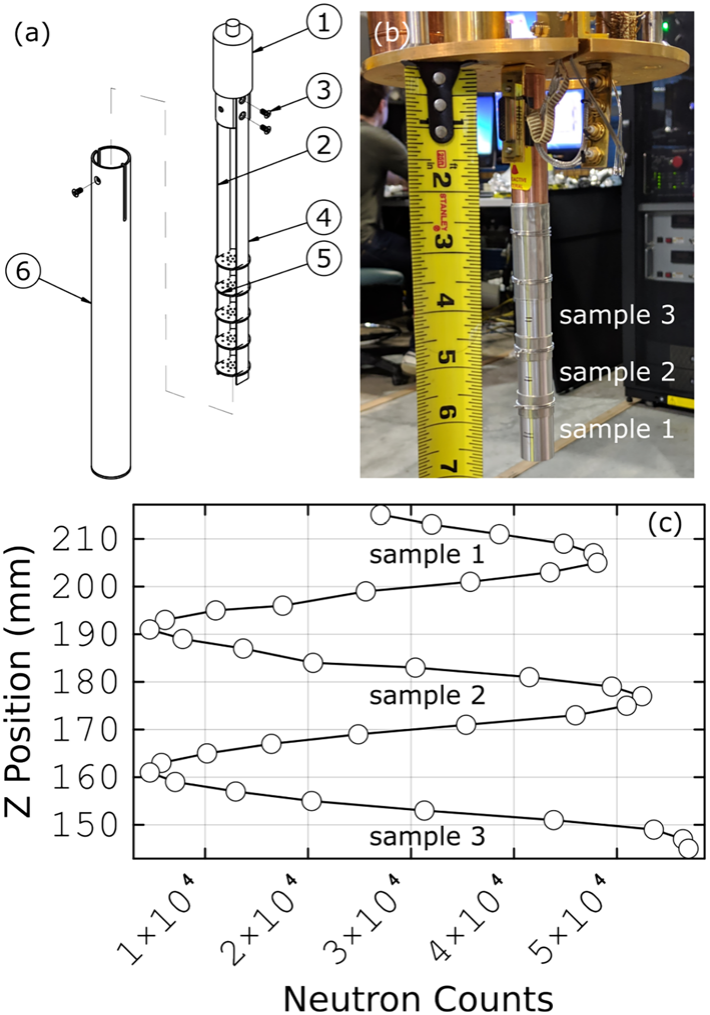
The CNCS single-crystal sample changer. (**a**) Schematic view and assembly of the sample changer. (1) sample holder head; (2,3) left and right legs; (4) rungs to hold individual crystals; (5) heat shield. (**b**) The sample changer loaded with three single crystals mounted on a 3He-4He dilution cryostat for ultra-low temperatures measurement. Numbers show the positions of three samples. Cadmium shielding in between the samples is visible. (**c**) The sample translation scan, variable *z*, shows three peaks due to incoherent elastic scattering from the crystals. It is used to determine the positions of the samples. at the start of an experiment.

Single crystal sample holders using the same vertical translation methodology have also been developed. Representative images of the multi-single-crystal changer are shown in Fig 14. The purpose of the changer is to accommodate multiple single-crystal samples simultaneously, and allow for changes between the samples at a given temperature by vertical translation. The sample changer is compatible with standard orange cryostats, CCR, or dilution refrigerators. A schematic view of the changer is shown in Fig 14a. The prototype of this sample changer was used for an experiment that was published in Ref^35^. The sample holder head, legs, and rungs are fabricated from OFHC copper alloy, enabling sample cooling to approximately 0.1 K. The heat shield is made of aluminum alloy. Single-crystal samples are arranged on the rungs in a stacked manner and are selected for measurement through the vertical translation of the sample environment stage. The other side of the rungs is covered with cadmium to minimize sample *crosstalk* via the scattered beam. A focusing guide must be used to focus the beam at the sample position^34^. Figure 14b illustrates the sample changer loaded with three samples on the bottom-loading *Triton NANO* cryogen-free dilution refrigerator.^36^ The neutron scattering z-scan, Fig. 14c,demonstrates the typical ratio of the incoherent neutron signal at the sample positions and between them. It is used to determine the sample positions for subsequent measurements.

These advancements in vertically stacked sample holders have allowed for a better optimization of the time required for technical staff time and for the time allotted for the neutron scattering measurements. Specifically, there have been cases where experiments simply would not have been feasible to run without the usage of sample changers.

## Conclusions

The increase in neutron flux available at the Oak Ridge National Laboratory’s Spallation Neutron Source due to the Proton Power Upgrade has made the use of sample changers for direct geometry neutron chopper spectrometers an appropriate investment. We have described a bottom loading and a top loading version of three sample changers that can be used at the flange mount style instruments at the facility. The changers described here can interchangeably be used on both the ARCS and the SEQUOIA instruments. Future development of the ARCS top-loading sample changer includes the addition of a vertical translation stage of the stick. This would allow for a second carousel of three samples to be available on the sample changer. If the double powder cans described above for CNCS are used in this configuration, the ARCS three-sample changer will be able to then accommodate twelve powder samples.
